# Supplementary material for: Resilience Interventions Conducted in Western and Eastern Countries—A Systematic Review
Source: Int J Environ Res Public Health. 2022 Jun 5;19(11):6913. doi: 10.3390/ijerph19116913 (PMC9180776; doi:10.3390/ijerph19116913)
Supplement: Supplementary file 1 [file ijerph-19-06913-s001.zip › Table S1.pdf]

Table S1. Search terms and syntax that were used in MEDLINE and adapted to use in other databases [26].

1 Resilience, Psychological/
2 social adjustment/
3 Adaptation, Psychological/
4 (post-traumatic growth or posttraumatic growth or stress-related growth).tw,kf.
5 (positiv$ adj1 (adapt$ or adjust$)).tw,kf.
6 (psychol$ adj1 (adapt$ or adjust$)).tw,kf.
7 (resilien$ or hardiness$).tw,kf.
8 (cope or coping).tw,kf.
9 ((withstand$ or overcom$ or resist$ or recover$ or thriv$ or adapt$ or adjust$ or bounc$ back) adj5 (stress$ or trauma$ or adversit$)).tw,kf.
10 or/1-9
11 exp psychotherapy/
12 Stress, Psychological/th
13 (psychotherap$ or psycho-therap$).tw,kf.
14 (behav$ adj3 (intervention$ or program$ or therap$)).tw,kf.
15 ((cognit$ or cognitive behavior$ or CBT) adj3 (intervention$ or program$ or therap$)).tw,kf.
16 (psycho$ adj3 (intervention$ or program$ or therap$)).tw,kf.
17 relaxation.tw,kf.
18 mindful$.tw,kf.
19 (counsel?ing or coaching).tw,kf.
20 (third wave adj (psycho$ or therap$)).tw,kf.
21 cognit$ restructur$.tw,kf.
22 positive psychology.tw,kf.
23 (refram$ or re-fram$ or reapprais$).tw,kf.
24 (stress adj1 (inoculation or manag$ or reduc$ or resist$)).tw,kf.
25 (anxiety adj3 manage$).tw,kf.
26 “acceptance and commitment ”.tw,kf.
27 Combined Modality Therapy/

28 (multimodal or multi-modal or combined modal$).tw,kf.

29 exp Health promotion/

30 (health adj3 (educat$ or promot$)).tw,kf.

31 or/11-30

32 10 and 31

33 (resilien$ adj5 (train$ or program$ or intervention$ or promot$ or prevent$ or enhanc$ or learn$ or teach$ or educat$ or increas$

or develop$ or manag$ or therap$ or protocol$ or treat$)).tw,kf.

34 (hardiness$ adj5 (train$ or program$ or intervention$ or promot$ or prevent$ or enhanc$ or learn$ or teach$ or educat$ or increas$

or develop$ or manag$ or therap$ or protocol$ or treat$)).tw,kf.

35 or/32-34

36 randomized controlled trial.pt.

37 controlled clinical trial.pt.

38 randomi#ed.ab.

39 placebo$.ab.

40 drug therapy.fs.

41 randomly.ab.

42 trial.ab.

43 groups.ab.

44 or/36-43

45 exp animals/ not humans.sh.

46 44 not 45

47 35 and 46

48 limit 47 to yr="1990 -Current"
