# Supplementary material for: Resilience Interventions Conducted in Western and Eastern Countries—A Systematic Review
Source: Int J Environ Res Public Health. 2022 Jun 5;19(11):6913. doi: 10.3390/ijerph19116913 (PMC9180776; doi:10.3390/ijerph19116913)
Supplement: Supplementary file 1 [file ijerph-19-06913-s001.zip › Table S2.pdf]

Table S2. Search terms and syntax that were used for a follow-up search in June 2019 in MEDLINE and adapted to use in other databases [12,13].

1 Resilience, Psychological/
2 social adjustment/
3 Adaptation, Psychological/
4 (post-traumatic growth or posttraumatic growth or stress-related growth).tw,kf
5 (positiv$ adj1 (adapt$ or adjust$)).tw,kf.
6 (psychol$ adj1 (adapt$ or adjust$)).tw,kf.
7 (resilien$ or hardiness$).tw,kf.
8 (cope or coping).tw,kf.
9 ((withstand$ or overcom$ orresist$ orrecover$ orthriv$ or adapt$ or adjust$ or bounc$ back) adj5 (stress$ ortrauma$ or adversit$)).tw,kf.
10 or/1-9
11 exp psychotherapy/
12 Stress, Psychological/th
13 (psychotherap$ or psycho-therap$).tw,kf.
14 (behav$ adj3 (intervention$ or program$ or therap$)).tw,kf.
15 ((cognit$ or cognitive behavior$ or CBT) adj3 (intervention$ or program$ or therap$)).tw,kf.
16 (psycho$ adj3 (intervention$ or program$ or therap$)).tw,kf.
17 relaxation.tw,kf.
18 mindful$.tw,kf.
19 (counsel?ing or coaching).tw,kf.
20 (third wave adj (psycho$ or therap$)).tw,kf.
21 cognit$ restructur$.tw,kf.
22 positive psychology.tw,kf.
23 (refram$ or re-fram$ or reapprais$).tw,kf.
24 (stress adj1 (inoculation or manag$ or reduc$ or resist$)).tw,kf.
25 (anxiety adj3 manage$).tw,kf.
26 "acceptance and commitment ".tw,kf.
27 Combined Modality Therapy/

28 (multimodal or multi-modal or combined modal$).tw,kf.

29 exp Health promotion/

30 (health adj3 (educat$ or promot$)).tw,kf.

31 or/11-30

32 10 and 31

33 (resilien$ adj5 (train$ or program$ or intervention$ or promot$ or prevent$ or enhanc$ or learn$ or teach$ or educat$ or increas$ or

develop$ or manag$ or therap$ or protocol$ or treat$)).tw,kf.

34 (hardiness$ adj5 (train$ or program$ or intervention$ or promot$ or prevent$ or enhanc$ or learn$ or teach$ or educat$ or increas$ or

develop$ or manag$ or therap$ or protocol$ or treat$)).tw,kf.

35 or/32-34

36 randomized controlled trial.pt.

37 controlled clinical trial.pt.

38 randomi#ed.ab.

39 placebo$.ab.

40 drug therapy.fs.

41 randomly.ab.

42 trial.ab.

43 groups.ab.

44 or/36-43

45 exp animals/ not humans.sh.

46 44 not 45

47 35 and 46

48 Health personnel/

49 (health$ adj3 (personnel or profession$ or worker$ or practitioner$ or provider$ or staI)).tw,kf.

50 ((medical care adj3 (personnel or profession$ or worker$ or practitioner$ or provider$ or staI)) or(medical adj3 (personnel or profession

$ or worker$ or practitioner$ or provider$ or staI))).tw,kf.

51 (care adj1 (personnel or profession$ or worker$ or practitioner$ or provider$ or staI)).tw,kf.

52 (doctor$ or physician$ or general practitioner$ or (primary care adj2 practitioner$) or surgeon$).tw,kf.

53 (nurse$ or (nursing adj3 assistant$) or (nursing adj3 staI)).tw,kf.

54 nursing.tw,kf

55 ((hospital or ambulance) adj1 personnel).tw,kf.

56 ((intensive adj2 care) or ICU or (intensive adj2 care adj2 unit adj3 personnel$)).tw,kf.

57 ((allied health$) adj2 (personnel or profession$ or worker$ or practitioner* or provider$ or staI)).tw,kf.

58 (psychologist$ or psychotherapist$ or psychiatrist$ or (mental health adj2 clinician$) or (mental health adj2 profession$) or (mental
health adj2 worker$)).tw,kf.

59 (social worker$).tw,kf.

60 (paramedic$ or ambulance or medic$ or ((first or emergency or disaster) adj1 (response or responder$))).tw,kf.

61 (professional adj1 (caregiver$ or care-giver$)).tw,kf.

62 ((physical therapist$) or physiotherapist$ or occupational therapist$ or recreational therapist$ or music therapist$ or art therapist$ or
dietitian$ or nutritionist$ or ((speech and language) adj1 therapist$) or speech pathologist$ or audiologist$ or exercise physiologist$ or
osteopath$ or sonographer$ orradiographer$ orradiotherapist$ or((radiology orradiation) adj1 (therapist$ ortechnician$ ortechnologist
$ or assistant$ or scientist$)) or respiratory therapist$ or ((anesthesia or anesthesiologist) adj1 (technician$ or assistant$)) or dental
hygienist$ or (surgical adj1 (technician$ or technologist$)) or orthotist$ or orthoptist$ or podiatrist$ or perfusionist$).tw,kf.

63 counsel?or$.tw,kf.

64 ((clinical or clinical laboratory or medical$ or medical$ laboratory) adj1 (technician$ or technologist$ or assistant$ or scientist$)).tw,kf.

65 ((human or health) adj1 service adj3 profession$).tw,kf.

66 (public health adj2 (service or agency)).tw,kf.

67 (secondary traumati?ation or (work$ adj2 (trauma survivor$))).tw,kf.

68 ((nursing or medical or premedical or paramedic or psychology or physical therapy or occupational therapy) adj2 student$).tw,kf.

69 (college adj2 student$).tw,kf.

70 ((nurs$ adj1 (graduate$ or education)) or (medic$ adj1 train$) or (student adj1 nurse$)).tw,kf.

71 or/48-70

72 47 and 71

73 limit 72 to yr="1990 -Current"

74 limit 73 to yr="2016 -Current"
