# Supplementary material for: Resilience Interventions Conducted in Western and Eastern Countries—A Systematic Review
Source: Int J Environ Res Public Health. 2022 Jun 5;19(11):6913. doi: 10.3390/ijerph19116913 (PMC9180776; doi:10.3390/ijerph19116913)
Supplement: Supplementary file 1 [file ijerph-19-06913-s001.zip › Table S3.pdf]

Table S3. Overview of all studies and the used psychological measures.[1]

Study	Resilience	Anxiety	Depression	Quality of Life	Perceived Stress	Social Support
Abbott (2009)		DASS-21	DASS-21	WHOQoL-BREF (psychological)	DASS-21	
Adler (2009)						
Adler (2015)						
Aikens (2014)	CD-RISC			Shirom Virgo Scale (cognitive liveliness)	PSS-14	
Akbari (2017)						
Almasi (2016)	CD-RISC					
Alschuler (2018)	CD-RISC	PROMIS	PROMIS	NeuroQoL		
Anshel (1990)						
Arch (2014)						
Arnetz (2009)					VAS	
Arnetz (2013)						
Ashing (2014)			CES-D			
Ashing (2016)				FACT-G		
Aubin (2011)						
Bahamin (2012)						
Bakhshizadeh (2016)	unspecified	GHQ-28	GHQ-28			
Baliousis (2016)	BRS	DASS-21	DASS-21		DASS-21	
Bari (2013)	CD-RISC					

Bartley (2016)						
Bauman (2015)				PWB		
Bavali (2013)	CD-RISC					
Beadel (2016)	RRT	ASI				
Bekki (2013)						
Bell (2017)	CD-RISC		PHQ-9	EurQol		
Berger (2011)			ProQoL			
Berger (2016)	CD-RISC					
Bernburg (2016)				unspecified	PSQ	
Betancourt (2014)						
Bian (2011)						Social Support Rating Scale (perceived support)
Botella (2016)		OASIS	BDI-II		PSS	
Botello (2015)		DASS-21	DASS-21	WHOQoL-BREF	DASS-21	
Bradshaw (2007)						
Broome 2011						
Brown (2016)			POMS	POMS	PSS	
Cacioppo (2015)						
Carlson (2013)				FACT-B	C-SOSI	MOS-SSS
Caruso (2014)		GAD-7	PHQ-9	QUAL-EC		
Castro (2006)						
Castro (2012)						

Cerezo (2014)	CD-RISC			SLS		
Chan (2012)		STAI				
Chandler (2015)	RS-14					
Chesak (2015)		GAD-7			PSS	
Cheung (2016)	CD-RISC			unspecified		MSPSS
Chongruksa (2015)	RS-14	GHQ-28	GHQ-28			
Christopher (2018)	CD-RISC	PROMIS	PROMIS		PSQ	
Chung (2013)	Subscales out of the BRS. RSCA. ARQ					
Church (2016)		SA-45	SA-45			
Cieslak (2016)			OLBI			
Rogala (2016)						
Creswell (2014)						
Deckro (2002)		STAI			PSS	
Devilly (2013)						
Duchemin (2015)				unspecified	PSS	
Erogul (2014)	RS-14				PSS-10	
Esmer (2010)						
Falb (2016)			PHQ-9			
Farchi (2010)						
Ferguson (2005)		DSP	DSP		DSP	
Foran (2012)						

Frye (2016)	BRS			SWWS	PSS-10	
Fuller-Tyszkiewicz (2017)	RS					MSPSS
Galante (2018)				WEMWBS	CORE-OM	
Gance-Cleveland (2008)						
Garland (2014)			C-SOSI			
Gaugler (2015)			CES-D		PSS	
Gelkopf (2008)						
Gerson (2013)	unspecified		BDI-II			
Geschwind (2011)			HDSR			
Geschwind (2015)						
Gonzalez (2006)					unspecified	
Grant (2009)	unspecified	DASS-21	DASS-21	WWBI	DASS-21	
Grant (2010)	CHS	DASS-21	DASS-21	WWBI	DASS-21	
Hadizad (2016)						
Haidarabadi (2014)						
Hallowell (2011)		DASS-21	DASS-21	WHOQoL-BREF (psychological)	DASS-21	
Hanna (2018)	RQS		ProQoL-V	GWS		
Hargrove (2012)						
Hawkes (2014)						
Haydari (2015)					PSS-14	
Heydarpour (2018)	CD-RISC			PWB		

Ho (2016)		HADS	HADS		PSS	
Hodges (2010)						
Hoge (2013)		BAI			SUDS during TSST	
Hourani (2011)						
Hourani (2016)						
Houston (2016)	CD-RISC	GAD-7	CES-D		items on 14 stressful experiences	
Hsiao (2012)			BDI-II			
Jennings (2002)				Index of Job Satisfaction		
Jennings (2013)			CES-D			
Jennings (2011)						
Jennings (2017)						
Jensen (2015)			MDI	WHO-5	PSS	
Johnson (2013)						
Kaboudi (2018)					Parental Stress Scale Short Form Questionnaire	
Kane (2016)						
Kanekar (2009)						ISEL
Kaveh (2011)				SWLS		
Kent (2011)		STAI	BDI-II	Psychological Well-being scale		
Khodabakhshi (2015)					DASS-21	
Klatt (2015)						

Klatt (2016)					PSS	
Kovacs (2012)		STAI-T	BDI (short version)	EORTC QLQ-C30 QoL		
Kovacs (2018)		HADS	HADS	SWLS		
Krabbenborg (2017)	RS-nl	BSI-53	BSI-53	Lehman Quality of Life Interview (short version)	BSI-18	
Kreutzer (2018)	CD-RISC				13 Items on Stress	
Lancer (2007)			CES-D			
Lantieri (2011)					PSS	
Patrao (2015)						
Liu (2008)		SAI	BDI-II	BMSWBI		
Loprinzi (2011)	CD-RISC	SAS		LASA	PSS	
Luthans (2008)						
Luthans (2010)						
Luthans (2014)						
Luthar (2000)			BDI			PCRI (parenting support)
Luthar (2017)			unspecified		PSI	
Luthar 2007						
Mache (2015)	unspecified			COPSOQ	PSQ	
Mache (2016)	BRCS			COPSOQ	PSQ	
Maddi (1998)				Measure of job satisfaction (Maddi. 1987)		Measure of social support (Moos. 1979)
May (2016)	CD-RISC			FACT-B		
McCann (2016)	RS					

McCraty (2012)		POQA	POQA		POQA	POQA
McGonagle (2014)	CD-RISC			3-item scale for job satisfaction (Cammann et al.)		
Mealer (2014)	unspecified	HADS				
Mejia-Downs (2016)					PSS-10	SPS
Melendez (2015)	BRCS					
Mistretta (2018)		DASS-21	DASS-21	WHO-5	DASS-21	
Moghimi (2017)	CD-RISC				PSI-SF	
Montgomery (2013)						
Mulligan (2011)						
Naemi (2015)	GHQ-28	GHQ-28	GHQ-28			
Nichols (2015)	CD-RISC	GAD-7	PHQ-9			
Norouzi (2017)						
O'Brien (2016)	CD-RISC					
O'Donnell (2013)	RS-14			SLS		
Oken (2016)			CES-D	SWLS	PSS	
Oman (2008)				PANAS	PSS	
Park (2009)						
Pauls (2016)	short version of Soucek et al. 2015					
Pawar (2016)	PRQ				PSS	
Perez-Blasco (2016)	BRSC	DASS	DASS		DASS	

Petree (2012)					PSS	
Pidgeon (2013)						
Pidgeon (2014)	unspecified				PSS	
Pietrowsky (2012)	RS-11		BDI-II	SWLS		
Porter (2008)		SCL-90-R				Peer Suppor Crisis Support Questionnaire (perceived peer support)
Poulsen (2015)						
Pyatak (2018)			PHQ-8	SWLS		
Rahmati (2017)	CD-RISC					
Ramos (2018)						ESSS
Ricelli (2016)	CD-RISC		PHQ-9		PHQ-15	
Rigby (2007)		HADS	HADS			
Roeser (2013)					measure created from items in other studies	
Rogerson (2016)	RAW					
Roghanchi (2013)	CD-RISC					
Rose (2013)					PSS-10	
Roshan (2015)	CD-RISC					
Ross (2014)	unspecified	DASS-21		WEMWBS	DASS-21	
Roustaei (2017)						
Roy (2016)						
Sadow (1993)						

Safarinia (2015)	CD-RISC					
Safren (2014)						
Sanchez-Teruel (2015)					PSI-SF	
Sansom-Daly (2013)						
Schachman (2004)				PWB		
Schotanus-Dijkstra (2017)	BRS	HADS	HADS	MHC-SF		
Schroeder (2016)	unspecified		MASL		PSS-10	
Seligman (2007)		BAI	BDI	SLC		
Shakespeare-Finch (2014)						
Sharma (2014)	CD-RISC	GAD-7		LASA	PSS	
Shochet (2011)						
Skeffington (2016)		DASS-21	DASS-21		DASS-21	SSQ (short version)
Smeets (2014)		PSWQ		SLS		
Sood (2011)		SAS		LASA	PSS	
Sood (2014)		SAS		LASA	PSS	
Sprange (2013)	BRS DRS		PHQ-9	EQ-5D-3 L		
Steinhardt (2008)	CD-RISC		CES-D		PSS	
Stephens (2012)	unspecified				PSS	SSS
Stoiber (2011)						
Strijk (2013)				RAND-36		
Tan (2016)	CD-RISC			WHOQoL		
Taylor (1997)						

Tierney (1997)						
Turkstra (2013)						
Tyson (2009)		Job-related anxiety-contentment scale (modified)	Job-related depression-enthusiasm scale (modified)			
van Berkel (2014)				EORTC QLQ-C30		
van der Spek (2017)		HADS	HADS	SPWB		
van Zelst (2010)						
Varker (2012)		DASS-21	DASS-21		DASS-21	ISEL-12
Victorson (2016)						
Villani (2013)		STAI-S				
Vranceanu (2016)		GAS-7	PHQ-9	WHOQoL-BREF (psychological)		
Vuori (2012)			BDI			
Waddell (2005)						
Waddell (2015)						
Wagner (2007)		BSI	BSI			
Waite (2004)	Spirit Core Scale (Innate resilience and reintegration)			employee satisfaction instrument (job satisfaction)		
Wakefield (2016)		DASS-21	DASS-21	Family Caregiver Tool Global Severity Index	DASS-21	
Wang (2012)	unspecified	SCL-90	SCL-90	GWS		
Weir (1997)				single item measure		
Weiss (2013)	BRS		CES-D	EQ-5D		

Weissberg-Benchell (2016)						
West (2014)			MBI		PSS	
West (2015)			PRIME-MD	LASA		
Wild (2016)	CD-RISC	GAD-7	PHQ-9	WEMWBS		13 Items on perceived social support (adapted from Sarason et al. 1987)
Wilson (2016)	RSES					
Wong (2009)						
Ye (2016)	CD-RISC	HADS	HADS	QLQ-C30		Social Support Scale
Ye (2017)		HADS	HADS	QLQ-C30		
Yu (2012)						
Yu (2014)						
Yuen (2015)						
Yun (2013)		HADS	HADS	SWLS EORTC QLQ-C30		
Yun (2017)		HADS	HADS	SWLS		MOS-SSS
Zautra (2008)						
Zernicke (2014)					CSOSI	
Zhang (2011)	RSA					
Zhang (2014)						
Zhang (2017)		STAI			CPSS	

[1] Beck Anxiety Inventory (BAI), Beck Depression Inventory (BDI-II), Body-Mind-Spirit Well-Being Inventory (BMSWBI), Brief Resilient Coping Scale (BRCS), Brief Resilience Scale (BRS), Connor-Davidson Resilience Scale (CD-RISC), Center for Epidemiologic Studies–Depression (CES-D), Coping Humor Scale (CHS), Copenhagen Psychosocial

Questionnaire (COPSOQ), Clinical Outcomes in Routine Evaluation Outcome Measure (CORE-OM), Child PTSD Symptom Scale (CPSS), Cognitive Style Indicator (CSOSI), Calgary Symptoms of Stress Inventory (C-SOSI), Calgary Symptoms of Stress Inventory (C-SOSI), Depression Anxiety and Stress Scale (DASS-21), Derogatis Psychological Testing (DSP), European Organisation for Research and Treatment of Cancer Quality of Life Questionnaire (EORTC QLQ C30), European Quality of Life Five Dimension (EQ-5D), European Quality of Life Five Dimension 3-Level (EQ-5D-3 L), Embodied Sense of Self Scale (ESSS), European Quality of Life (EurQoL), Functional Assessment of Cancer Therapy-Breast (FACT-B), Functional Assessment of Cancer Therapy-General (FACT-G), Generalized Anxiety Disorder 7-item scale (GAD-7), General Health Questionnaire 28-item scale (GHQ-28), General Well-Being Schedule (GWS), Hospital Anxiety and Depression Scale (HADS), Hamilton Depression Rating Scale (HDSR), Interpersonal Support Evaluation List (ISEL), Interpersonal Support Evaluation List 12-item scale (ISEL-12), Linear Analog Self Assessment Scale (LASA), The Maslach Burnout Inventory (MASL), Multiscore Depression Inventory (MDI), Mental Health Continuum-Short Form (MHC-SF), MOS Social Support Survey (MOS-SSS), Multidimensional Scale of Perceived Social Support (MSPSS), Neurology Quality of Life (NeuroQoL), Overall Anxiety Severity and Impairment Scale (OASIS), Oldenburg Burnout Inventory (OLBI), Positive Affect and Negative Affect Scales (PANAS), Parent-Child Relationship Inventory (PCRI), Patient Health Questionnaire 15-item scale (PHQ-15), Patient Health Questionnaire 9-item scale (PHQ-9), Profile of Mood States (POMS), Personal and Organizational Assessment (POQA), Primary Care Evaluation of Mental Disorders (PRIME-MD), Patient Reported Outcomes Measurement Information System (PROMIS), Professional Quality of Life scale (ProQoL), Parenting Stress Index-Short Form (PSI-SF), Parenting Stress Index-Short Form (PSI-SF), Perceived Stress Questionnaire (PSQ), Perceived Stress Questionnaire (PSQ), Perceived Stress Scale (PSS), Perceived Stress Scale 10-item scale (PSS-10), Perceived Stress Scale 14-item scale(PSS-14), Penn State Worry Questionnaire (PSWQ), Psychological Well-Being Scales (PWB), Quality of Life at the End of Life Cancer (QUAL-EC), RAND-36 vitality scale (RAND-36), Resilience at Work Scale (RAW), Romance Qualities Scale (RQS), Resilience-congruent recognition ratings (RRT), Resiliecne Scale 11-item scale (RS-11), Resilience Scale 14-item scale (RS-14), Resiliency Scale for Children and Adolescents (RSCA), Rosenberg Self-Esteem (RSES), Dutch version of the Resilience Scale (RS-nl), Symptom assessment-45 questionnaire (SA-45), Spiritual Assessment Inventory (SAI), Smith Anxiety Scale (SAS), Symptom Checklist 90 Revised (SCL-90-R), Satisfaction with Life Scale (SLS), Sensory processing sensitivity (SPS), Scales of Psychological Well-Being (SPWB), Social Support Questionnaire (SSQ short), Social Support Scale (SSS), State Trait Anxiety Inventory (STAI), State Trait Anxiety Inventory-Trait (STAI-T), Subjective Units of Distress Scale (SUDS), Trier Social Stress Tests (TSST), Satisfaction with Life Scale (SWLS), Satisfaction with Work Scale (SWWS), Visual Analog Scale (VAS), Warwick-Edinburgh Mental Well-Being Scale (WEMWBS), World Health Organisation-Five Well-Being Index (WHO-5), World Health Organization Quality of Life questionnaire (WHOQoL-BREF), Workplace Well-Being Index (WWBI).
