# Supplementary material for: Resilience Interventions Conducted in Western and Eastern Countries—A Systematic Review
Source: Int J Environ Res Public Health. 2022 Jun 5;19(11):6913. doi: 10.3390/ijerph19116913 (PMC9180776; doi:10.3390/ijerph19116913)
Supplement: Supplementary file 1 [file ijerph-19-06913-s001.zip › Table S4.pdf]

Table S4. Overview of the included studies in the meta-analysis for the outcome resilience.

Study	Resilience defined as state/trait	Stressor	non-/normative stressors	Participants in IG	Total number of participants randomized for IG and CG
Aikens (2014)	State	workplace employee stress	normative	44	89
Almasi (2016)	State	disabled children	non-normative	30	60
Bakhshizadeh (2016)	State	Slow Pace Children	non-normative	20	40
Baliousis (2016)	State	Haematopoietic stem cell transplantation (HSCT)	non-normative	21	45
Bavali (2013)	State	intellectual disability Children	non-normative	13	26
Bell (2017)	State	Mild traumatic brain injury (mTBI)	non-normative	178	356
Berger (2016)	State	educators who were working with elementary schoolchildren exposed to the Canterbury earthquake in New Zealand. Primary trauma (as a result of the disaster itself) and secondary trauma (as a result of working with traumatized students)	non-normative	35	69
Cerezo (2014)	State	breast cancer	non-normative	101	207
Cheung (2016)	State	disaster	non-normative	458	918
Chongruksa (2015)	State	Military unrest	non-normative	30	60
Christopher (2018)	State	stressful occupation law enforcement officers	non-normative	16	33
Erogul (2014)	State	stress in medical school	non-normative	39	81
Gerson (2013)	State	undergrade	normative	16	33
Grant (2009)	Trait	workplace related stressors	normative	20	50
Grant (2010)	Trait	workplace related stressors	normative	23	50
Hanna (2018)	State	working as a human service professionell	non-normative	25	50
Houston (2016)	State	undergraduated university students	normative	64	129
Krabbenborg (2017)	State	homelessness	non-normative	117	251
Kreutzer (2018)	State	challenges after a traumatic brain injury	non-normative	83	160
Mache (2015)	State	working as a surgeon	non-normative	36	69

Mache (2016)	State	working as a psychiatrist	non-normative	38	76
May (2016)	State	breast cancer	non-normative	13	22
McCann (2016)	State	depression	non-normative	27	54
McGonagle (2014)	State	chronic illness	non-normative	30	59
Mealer (2014)	State	stressful work, working as a nurse	non-normative	14	29
O'Donnell (2013)	State	physical disabilities	non-normative	21	41
Pauls (2016)	State	workrelated stressors	normative	57	113
Perez-Blasco (2016)	State	age-associated loss of resources, both material and personal	normative	22	45
Pidgeon (2014)	State	working as a human service professionell	non-normative	23	46
Ricelli (2016)	State	homeless or at-risk for homelessness	non-normative	31	51
Rogerson (2016)	State	workrelated stressors	normative	14	28
Ross (2014)	State	workrelated stressors	normative	49	100
Safarinia (2015)	State	academic stress	normative	17	34
Schotanus-Dijkstra (2017)	State	suboptimal mental health	non-normative	137	275
Schroeder (2016)	State	primary care physicians	non-normative	16	33
Steinhardt (2008)	State	academic stress	normative	31	64
Stephens (2012)	State	academic stress	normative	35	70
Tan (2016)	State	mental illness	non-normative	32	64
Waite (2004)	State	workrelated stressors	normative	123	232
Wang (2012)	State	mental crisis	non-normative	70	70
Weiss (2013)	State	social isolation, have health problems and a low socioeconomic status (SES)	non-normative	58	108
Wilson (2016)	State	military personell stressors	non-normative	149	282
Zhang (2011)	State	military and academic stressors	non-normative	43	125
