# Supplementary figures and images for: Resilience Interventions Conducted in Western and Eastern Countries—A Systematic Review
Source: Int J Environ Res Public Health. 2022 Jun 5;19(11):6913. doi: 10.3390/ijerph19116913 (PMC9180776; doi:10.3390/ijerph19116913)

Figure S2. Forest plot for anxiety.

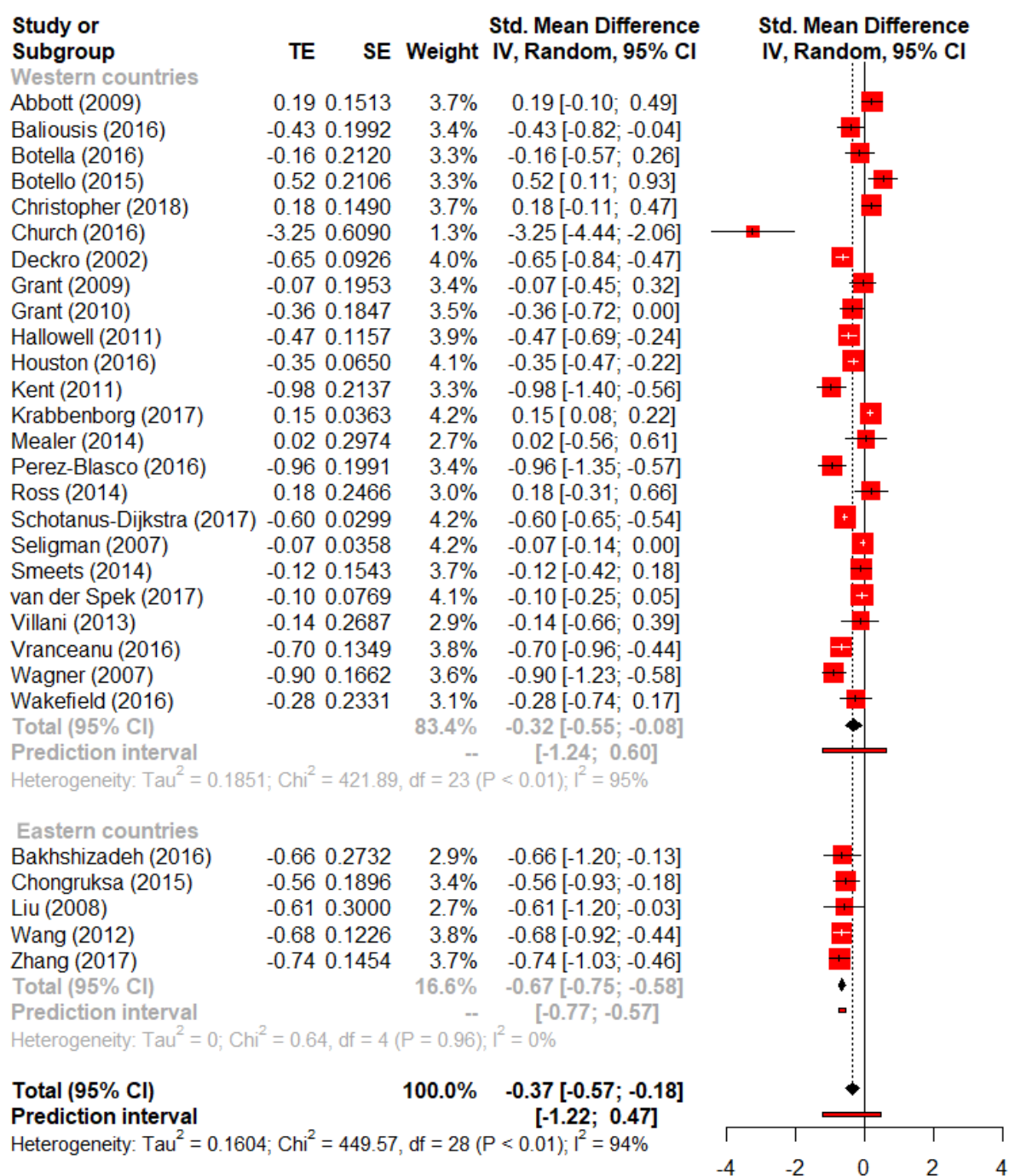

Supplement: Supplementary file 1 [file ijerph-19-06913-s001.zip › Figure S2.pdf]

**Figure S3.** Forest plot for depressive symptoms.

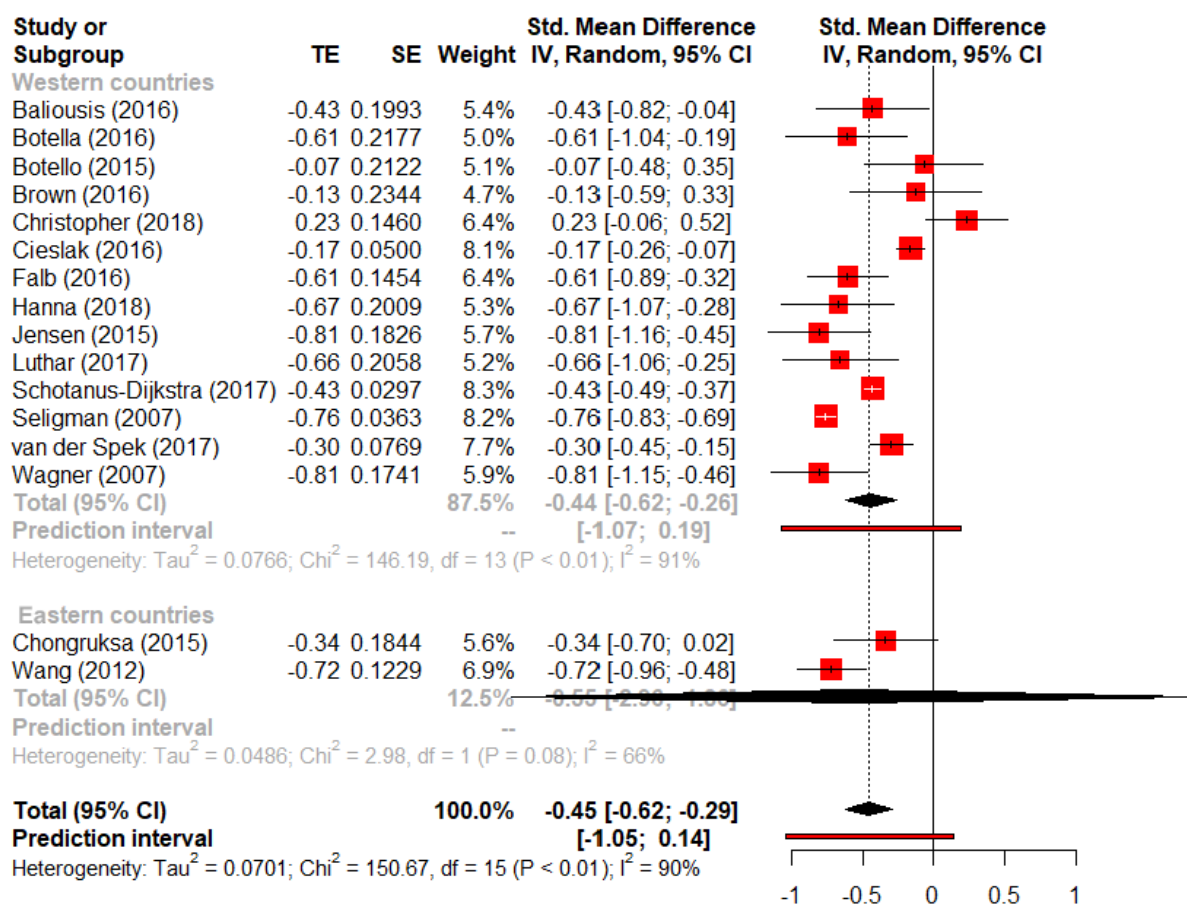

Supplement: Supplementary file 1 [file ijerph-19-06913-s001.zip › Figure S3.pdf]

Figure S4. Forest plot for quality of life.

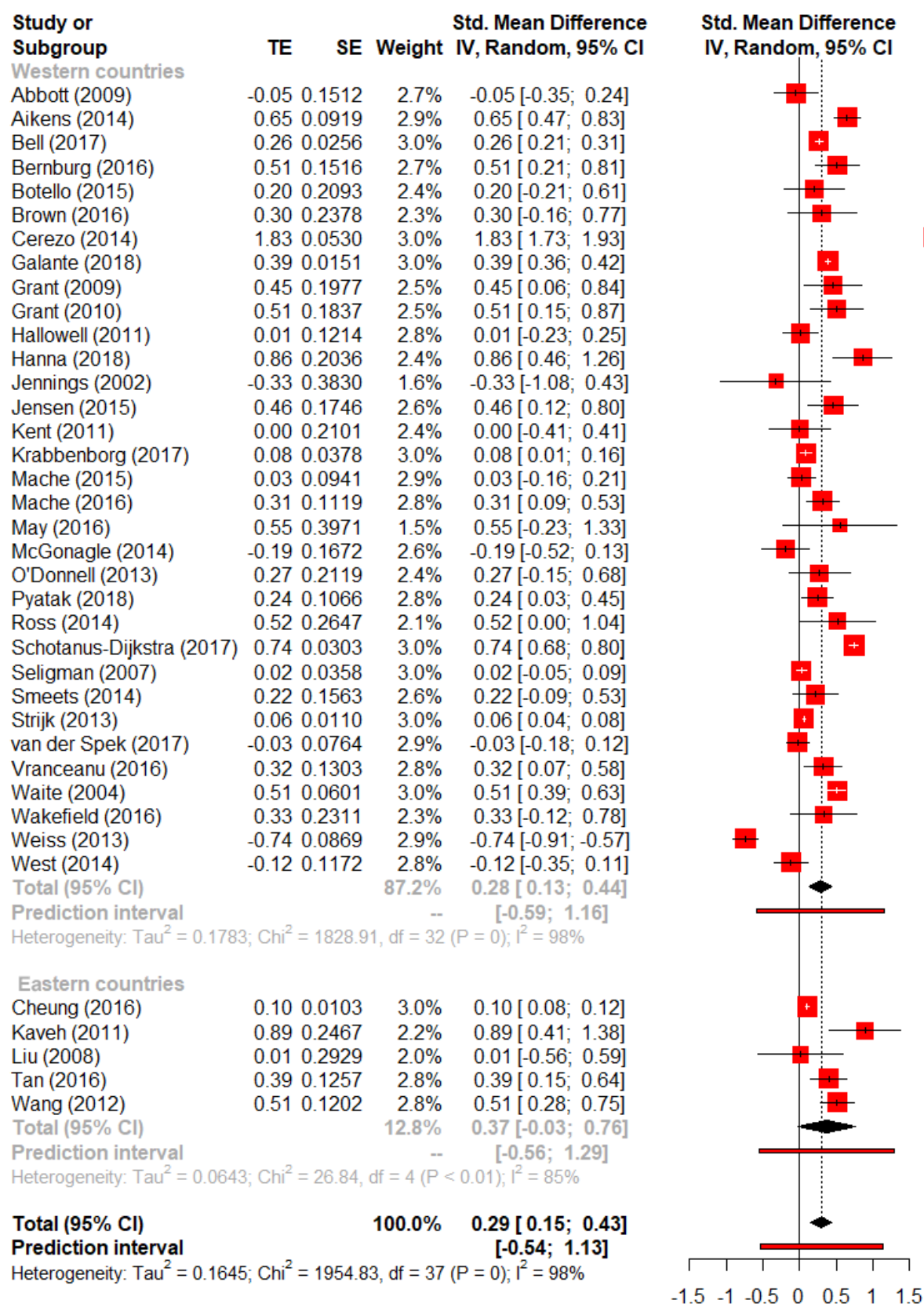

Supplement: Supplementary file 1 [file ijerph-19-06913-s001.zip › Figure S4.pdf]

Figure S5. Forest plot for perceived stress.

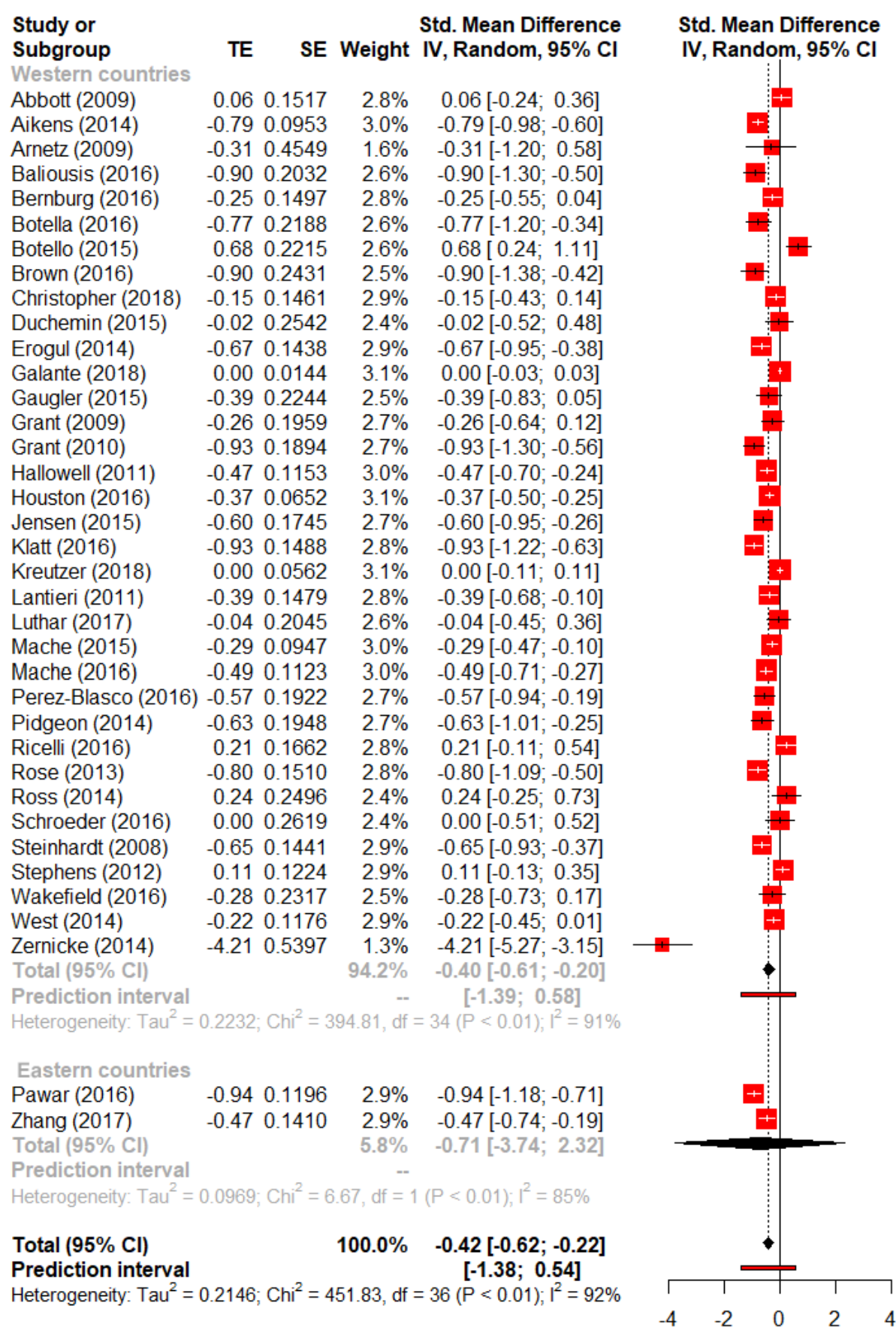

Supplement: Supplementary file 1 [file ijerph-19-06913-s001.zip › Figure S5.pdf]

**Figure S6.** Forest plot for social support.

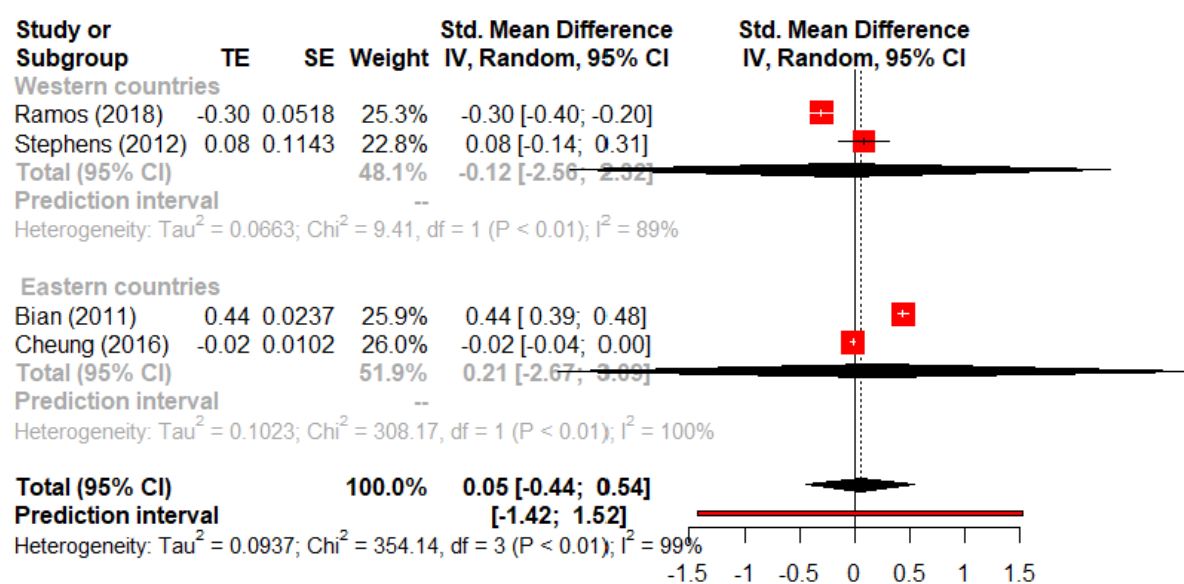

Supplement: Supplementary file 1 [file ijerph-19-06913-s001.zip › Figure S6.pdf]
