# Supplementary material for: Resilience Interventions Conducted in Western and Eastern Countries—A Systematic Review
Source: Int J Environ Res Public Health. 2022 Jun 5;19(11):6913. doi: 10.3390/ijerph19116913 (PMC9180776; doi:10.3390/ijerph19116913)
Supplement: Supplementary file 1 [file Figure S1.pdf › Figure S1.pdf]

Figure S1. Risk of Bias Summary.

Study	1. Random sequence generation (selection bias)	2. Allocation concealment (selection bias)	3. Blinding of participants and personnel (performance bias): Objective outcomes	4. Blinding of participants and personnel (performance bias): Subjective outcomes	5. Blinding of outcome assessors (detection bias): Objective outcomes	6. Blinding of outcome assessors (detection bias): Subjective outcomes	7. Incomplete outcome data (attrition bias)	8. Selective outcome reporting (reporting bias)
Abbott 2009	?	?	+	−	+	?	+	−
Adler 2009	?	?	+	−	+	?	+	−
Adler 2015	?	?	+	−	+	?	−	+
Aikens 2014	+	?	+	−	+	?	+	+
Akbari 2017	?	?	+	−	+	?	?	−
Almasi 2016	?	?	+	−	+	?	?	+
Alschuler 2018	+	?	+	−	+	?	−	−
Anshel 1990	?	?	+	−	+	?	?	+
Arch 2014	?	?	+	−	+	?	?	+
Arnetz 2009	?	?	+	−	+	?	?	−
Arnetz 2013	?	?	+	−	+	?	−	−
Ashing 2014	+	?	+	−	+	?	?	+
Ashing 2016	+	?	+	−	+	?	−	+
Aubin 2011	?	?	?	?	?	?	?	?
Bahamin 2012	?	?	+	?	+	?	?	+
Bakhshizadeh 2016	?	?	+	−	+	?	−	+
Baliousis 2016	+	?	+	−	+	−	−	−
Bari 2013	?	?	+	−	+	?	?	+
Bartley 2016	?	?	?	?	?	?	?	?
Bauman 2015	+	?	+	−	+	?	−	+
Bavali 2013	?	?	+	−	+	?	?	+
Beadel 2016	?	?	+	?	+	?	−	−
Bekki 2013	−	−	+	?	+	?	−	+
Bell 2016	+	+	+	−	+	+	−	−
Berger 2011	+	?	+	−	+	?	?	+
Berger 2016	−	?	+	−	+	?	−	+
Bernburg 2016	+	?	+	−	+	?	?	+
Betancourt 2014	+	?	+	−	+	+	+	−
Bian 2011	?	?	+	−	+	?	−	+
Botella 2016	?	?	+	−	+	?	−	+
Botello 2015	+	?	+	−	+	?	+	+
Bradshaw 2007	−	−	+	−	+	?	−	?
Broome 2011	?	?	+	−	+	?	?	+
Brown 2016	?	?	+	−	+	?	+	+
Cacioppo 2015	?	?	+	−	+	?	−	+
Carlson 2013	+	?	+	−	+	?	−	+
Caruso 2014	?	?	+	−	+	?	?	−
Castro 2006	?	?	?	?	?	?	?	?
Castro 2012	?	?	+	−	+	?	−	+
Cerezo 2014	+	?	+	−	+	?	−	+
Chan 2012	+	?	+	−	+	?	−	+
Chandler 2015	+	?	+	−	+	?	+	+
Chesak 2015	+	?	+	−	+	?	−	+
Cheung 2016	+	?	+	−	+	?	−	+
Chongruksa 2015	?	?	+	−	+	?	−	+
Christopher 2018	?	?	+	−	+	?	−	−
Chung 2013	?	?	+	−	+	?	?	+
Church 2016	+	?	+	−	+	?	+	−
Cieslak 2016	?	?	+	?	+	?	−	−
Creswell 2014	?	?	+	?	+	?	−	+
Deckro 2002	?	?	+	−	+	−	−	+
Devilly 2013	?	?	+	−	+	?	−	−
Erogul 2014	?	?	+	−	+	?	−	+
Esmer 2010	?	?	+	−	+	?	−	+
Falb 2016	?	?	+	−	+	?	−	+
Farchi 2010	?	?	+	−	+	?	?	−
Ferguson 2005	?	?	+	−	+	?	−	+
Foran 2012	?	?	+	−	+	?	?	+
Frye 2016	+	?	+	−	+	?	−	−
Fuller Tyszkiewicz 2017	?	?	+	?	+	?	−	−
Galante 2018	+	?	+	−	+	+	−	−
Gance Cleveland 2008	+	?	+	−	+	+	−	−
Garland 2014	+	+	+	−	+	+	+	?
Gaugler 2015	+	?	+	−	+	−	?	+
Gelkopf 2008	+	?	+	−	+	?	−	+
Gerson 2013	+	?	+	−	+	?	−	+
Geschwind 2011	+	+	+	−	+	−	+	−
Geschwind 2014	?	?	+	−	+	?	?	+
Gonzalez 2006	?	?	?	?	?	?	?	?
Grant 2009	?	?	+	−	+	?	−	+
Grant 2010	?	?	+	−	+	?	−	+
Hadizad 2016	?	?	+	−	+	?	?	+
Haidarabadi 2014	?	?	+	−	+	?	?	+
Hallowell 2011	?	?	+	?	+	?	−	−
Hanna 2018	+	?	+	−	+	?	−	+
Hargrove 2012	?	?	+	−	+	?	−	+
Hawkes 2014	+	?	+	−	+	?	−	−
Haydari 2015	?	?	+	−	+	?	?	−
Heydarpour 2018	?	?	+	−	+	?	?	?
Ho 2013	+	?	+	−	+	?	+	+
Hodges 2010	?	?	+	−	+	?	−	+
Hoge 2013	?	?	+	−	+	?	−	−
Hourani 2011	?	?	+	+	+	+	?	−
Hourani 2016	?	?	+	−	+	?	−	+
Houston 2016	?	?	+	−	+	?	−	+
Hsiao 2012	+	?	+	−	+	?	−	+
Jennings 2002	−	?	+	−	+	?	−	+
Jennings 2011	?	?	+	−	+	?	−	−
Jennings 2013	?	?	+	−	+	?	−	+
Jennings 2017	+	?	+	−	+	?	+	+
Jensen 2015	+	+	+	−	+	?	+	−
Johnson 2013	?	?	+	−	+	?	+	+
Kaboudi 2018	−	−	+	−	+	?	?	−
Kane 2016	?	?	+	−	+	?	?	+
Kanekar 2010	?	?	+	?	+	?	−	+
Kaveh 2011	?	?	+	−	+	?	?	+
Kent 2011	?	?	+	−	+	?	+	+
Khodabakhshi 2015	?	?	+	−	+	?	?	+
Klatt 2015	?	?	+	−	+	?	?	+
Klatt 2016	+	?	+	−	+	?	−	−
Kovacs 2015	+	?	+	−	+	?	−	−
Kovacs 2018	?	?	+	−	+	?	−	−
Krabbenborg 2017	?	?	+	−	+	?	+	−
Kreutzer 2018	?	?	+	−	+	?	−	−
Lancer 2007	−	−	+	−	+	?	−	+
Lantieri 2011	?	?	+	−	+	?	−	+
Liu 2008	+	?	+	−	+	−	−	+
Loprinzi 2011	+	?	+	−	+	?	−	+
Luthans 2008	+	?	+	?	+	?	?	+
Luthans 2010	?	?	+	−	+	?	?	+
Luthans 2014	?	?	+	−	+	?	?	+
Luthar 2000	?	?	+	−	+	?	−	+
Luthar 2007	+	?	+	−	+	?	+	−
Luthar 2017	?	?	+	−	+	?	−	−
Mache 2015	+	?	+	−	+	?	+	+
Mache 2016	+	?	+	−	+	?	−	+
Maddi 1998	−	?	+	−	+	?	−	+
May 2016	?	?	+	−	+	?	?	+
McCann 2016	+	+	+	−	+	−	+	−
McCraty 2012	?	?	+	−	+	−	−	+
McGonagle 2014	+	?	+	−	+	?	−	+
Mealer 2014	?	?	+	−	+	?	−	+
Mejia Downs 2016	?	?	+	−	+	?	+	+
Melendez 2015	?	?	+	−	+	?	?	−
Mistretta 2018	?	?	+	−	+	?	+	−
Moghimi 2017	?	?	+	−	+	?	?	+
Montgomery 2013	+	?	+	−	+	−	−	−
Mulligan 2011	−	−	+	−	+	?	+	+
Naemi 2015	?	?	+	−	+	?	?	+
Nichols 2016	?	?	?	?	?	?	?	?
Norouzi 2017	?	?	+	−	+	?	−	−
OBrien 2016	+	?	+	−	+	?	−	−
ODonnell 2013	−	−	+	−	+	?	−	+
Oken 2016	+	?	+	−	+	+	−	−
Oman 2008	−	?	+	−	+	?	−	+
Park 2009	+	?	+	−	+	?	?	+
Patrao 2015	?	?	?	?	?	?	?	?
Pauls 2016	?	?	+	+	+	?	−	+
Pawar 2016	?	?	+	−	+	?	?	+
Perez Blasco 2016	+	?	+	−	+	−	−	−
Petree 2012	?	?	+	−	+	?	?	+
Pidgeon 2013	?	?	?	?	?	?	?	?
Pidgeon 2014	?	?	+	−	+	?	−	+
Pietrowsky 2012	?	?	+	−	+	?	+	+
Porter 2008	?	?	+	−	+	?	−	+
Poulsen 2015	+	?	+	−	+	?	−	+
Pyatak 2018	+	?	+	−	+	+	−	+
Rahmati 2017	?	?	+	−	+	?	?	+
Ramos 2018	?	?	+	−	+	?	−	−
Ratna 2015	?	?	+	?	+	?	−	+
Ricelli 2016	−	−	+	−	+	−	+	+
Rigby 2007	?	?	+	−	+	?	−	+
Roeser 2013	?	?	+	−	+	?	−	+
Rogala 2016	?	?	?	?	?	?	?	?
Rogerson 2016	+	?	+	−	+	?	−	+
Roghanchi 2013	?	?	+	−	+	?	?	+
Rose 2013	?	?	+	−	+	?	−	+
Roshan 2015	?	?	+	−	+	?	?	?
Ross 2015	+	?	+	+	+	+	−	+
Roustaei 2017	?	?	+	?	+	?	?	+
Roy 2015	+	?	+	−	+	?	−	−
Sadow 1993	?	?	+	−	+	?	?	−
Safarinia 2015	?	?	+	−	+	?	?	+
Safren 2014	+	?	+	−	+	+	−	−
Sanchez Teruel 2015	?	?	+	−	+	?	−	+
Sansom Daly 2013	?	?	?	?	?	?	?	?
Schachman 2004	+	?	+	−	+	?	−	−
Schotanus Dijkstra 2017	+	?	+	−	+	?	+	+
Schroeder 2016	?	?	?	?	−	−	−	+
Seligman 2007	?	?	+	−	+	?	−	+
Shakespeare Finch 2014	+	?	+	−	+	?	−	+
Sharma 2014	+	?	+	−	+	?	+	+
Shochet 2011	?	?	+	?	+	?	?	+
Skeffington 2016	+	+	+	−	+	?	−	−
Smeets 2014	+	?	+	−	+	?	−	+
Sood 2011	?	?	+	−	+	?	−	+
Sood 2014	?	?	+	−	+	?	+	+
Sprange 2013	+	+	+	−	+	−	−	+
Steinhardt 2008	?	?	+	−	+	?	−	−
Stephens 2012	+	?	+	−	+	−	?	+
Stoiber 2011	?	?	+	−	+	?	?	+
Strijk 2013	+	+	+	−	+	?	+	−
Tan 2016	+	?	+	−	+	−	+	−
Taylor 1997	?	?	+	−	+	?	?	+
Tierney 1997	?	?	+	−	+	?	?	+
Turkstra 2013	?	?	+	−	+	?	−	−
Tyson 2009	?	?	+	−	+	?	+	+
vanBerkel 2014	+	?	+	−	+	?	+	−
VanDerSpek 2017	+	?	+	−	+	?	−	−
VanZelst 2010	?	?	?	?	?	?	?	?
Varker 2012	?	?	+	−	+	?	−	−
Victorson 2016	+	?	+	−	+	?	?	−
Villani 2013	?	?	+	?	+	?	+	+
Vranceanu 2016	+	?	+	−	+	?	+	−
Vuori 2012	+	+	+	−	+	?	−	+
Waddell 2005	?	?	+	−	+	?	?	+
Waddell 2015	+	?	+	−	+	?	−	?
Wagner 2007	+	?	+	−	+	?	−	−
Waite 2004	?	?	+	−	+	?	−	+
Wakefield 2016	+	+	+	−	+	?	−	+
Wang 2012	?	?	+	−	+	?	−	+
Weir 1997	+	?	+	?	+	?	−	−
Weiss 2013	+	?	+	−	+	−	+	+
Weissberg Benchell 2016	?	+	+	−	+	?	?	?
West 2014	+	?	+	−	+	?	−	−
West 2015	?	?	+	−	+	?	?	+
Wild 2016	?	?	+	−	+	?	−	+
Wilson 2016	?	?	+	−	+	?	−	+
Wong 2009	−	?	+	−	+	?	?	?
Ye 2016	?	?	+	−	+	?	−	+
Ye 2017	+	+	+	−	+	+	−	+
Yu 2012	?	?	?	?	?	?	?	?
Yu 2014	+	+	+	−	+	?	−	+
Yuen 2015	+	?	?	−	?	+	?	?
Yun 2013	?	?	+	−	+	?	+	−
Yun 2017	+	?	+	−	+	?	+	−
Zautra 2008	+	?	+	−	+	?	−	−
Zernicke 2014	+	?	+	−	+	?	+	+
Zhang 2011	?	?	+	−	+	?	−	+
Zhang 2014	?	?	+	−	+	?	−	+
Zhang 2017	+	?	+	−	+	+	−	+
